# A novel fully human monoclonal antibody that neutralizes multiple human IFN-α subtypes effectively: a candidate therapy for SLE

**DOI:** 10.1186/2051-1426-3-S2-P240

**Published:** 2015-11-04

**Authors:** Shuang Wang, Peng Du

**Affiliations:** 1 Institute of Biotechnology, Academy of Military Medical Sciences, Beijing, People's Republic of China

## 

Systemic lupus erythematosus is a chronic, heterogeneous autoimmune disease, and there is no specific drug for its effective treatment, which may be because of its complexity on pathogenic mechanism. A multitude of studies of SLE in the last decade have accentuated a central role of the interferon-alpha (IFN-α) pathway in SLE pathogenesis. And the Clinical Trials have proved the effectiveness of antibodies targeting multiple human IFN-α subtypes, especially the sifalimumab of AstraZeneca (IIb). A novel fully human antibody that neutralizes multiple human IFN-α subtypes effectively was screened from fully synthetic human antibody library by Institute of Biotechnology, Academy of Military Medical Sciences. The antibody could effectively neutralize all the 12 subtypes except of IFN-α7, and its epitope is thoroughly distinct from others on research, which made its neutralizing spectrum different, and neutralizing efficacy stronger.

